# 3R-WS_2_ crystals as a breakthrough in compact entangled photon sources

**DOI:** 10.1038/s41377-024-01688-8

**Published:** 2025-02-27

**Authors:** Xinyu Yang, Mengxi Tan, David J. Moss

**Affiliations:** 1 School of Electronic and Information Engineering, Beihang University, Beijing, 100191 China; 2 Optical Sciences Centre, Swinburne University of Technology, Hawthorn, VIC 3122 Australia

**Keywords:** Optics and photonics, Optical physics

## Abstract

In a breakthrough that promises to revolutionize quantum photonic systems, researchers have successfully demonstrated a high-performance, ultracompact polarization-entangled photon-pair source using the van der Waals-based two-dimensional 3R-WS_2_ crystal. This achievement opens new avenues for integrated quantum technologies, paving the way for advanced applications in quantum computing, communication, and metrology.

Quantum entanglement is a cornerstone of quantum information science, playing a critical role in quantum computing, cryptography, and sensing^1^. Entangled photon pairs, in particular, have been instrumental in experiments demonstrating quantum nonlocality, such as Bell’s inequality violations. These entangled photons are essential components of quantum communication networks, quantum key distribution, and photonic quantum circuits^2^.

Traditionally, spontaneous parametric down-conversion (SPDC) in nonlinear crystals has been the method of choice for generating entangled photon pairs. However, these sources are often bulky and difficult to integrate into compact quantum photonic circuits. As the field of quantum technologies moves toward miniaturization and integration, the demand for smaller, more efficient sources of entangled photons has grown^3^. This is where van der Waals (vdW) materials, particularly the 3 R polytype of tungsten disulfide (WS_2_), come into play (Fig. 1).

**Fig. 1:**
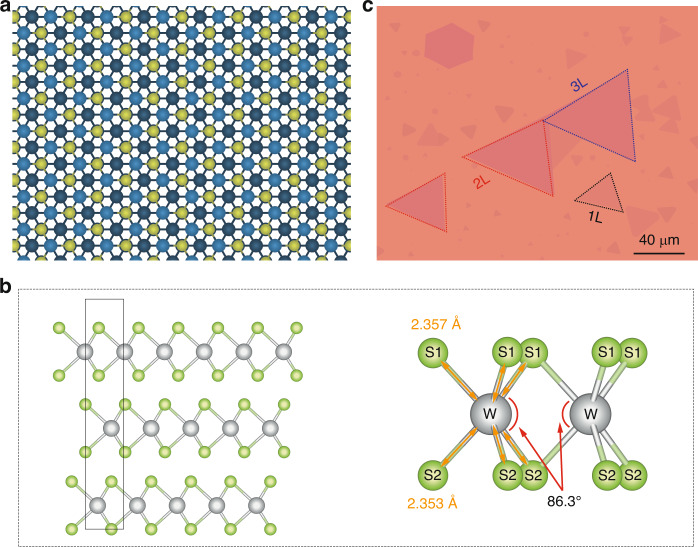
Structure of 3R-WS2 crystal^6–8^. (**a**) Top view of trilayer 3R-WS_2_ structure (**b**) 3R-WS_2_ crystal structure (left) and bond details between W and S atoms in monolayer (**c**) Optical micrograph of 3R-WS_2_ sample, with 1 L, 2 L, and 3 L representing monolayer, bilayer, and trilayer respectively

Van der Waals materials have attracted significant interest in recent years due to their unique optical and electronic properties. These materials exhibit strong excitonic effects, large nonlinear susceptibilities, and subwavelength thicknesses, making them ideal candidates for integrated photonic devices. In addition, vdW materials are compatible with a wide range of nanophotonic components, enabling the integration of vdW photon sources with existing quantum photonic architectures and allowing for the generation of more complex entangled states generated in a compact footprint^3^.

One of the key advantages of vdW materials like 3R-WS_2_ is their ability to maintain broken centrosymmetry even in ultrathin layers, which is crucial for efficient SPDC, as it allows for the scaling of optical nonlinearity with crystal thickness^4^. In contrast, many other vdW materials, such as the 2H polytype of WS_2_, lose their nonlinear properties in multilayer structures due to centrosymmetry restoration^5^. While other materials, such as 3R-MoS_2_, have been explored for photon-pair generation, the photon-pair generation rate in 3R-WS_2_ is over 200 times higher than that of 3R-MoS_2_, which suffers from photoluminescence noise and lower second-order nonlinear susceptibility^4^. The higher photon-pair generation rate of 3R-WS_2_, combined with its high coincidence-to-accidental ratio (CAR) and fidelity, makes it a superior choice for quantum photonic applications.

Miniaturizing quantum devices requires compact, high-fidelity sources of entangled photon pairs that can be integrated with nanophotonic structures. The critical requirements for these sources include well-defined quantum entanglement, high photon-pair generation rates, and scalability of optical nonlinearity. Achieving all these goals simultaneously in a compact device has been a major challenge for researchers.

The breakthrough reported by Feng et al. in the journal “*eLight*” addresses these challenges using 3R-WS_2_, a vdW material that exhibits remarkable optical properties and nonlinearities. By leveraging the unique lattice symmetry and second-order optical susceptibility of 3R-WS_2_, the researchers have achieved high-purity polarization entanglement in a compact form factor, demonstrating an ultracompact photon-pair source with a high CAR and exceptional fidelity.

Feng et al.’s work focuses on the use of 3R-WS_2_, a layered material with broken centrosymmetry and three-fold rotational symmetry. These properties are crucial for achieving efficient second-order nonlinear optical processes such as SPDC. The researchers successfully demonstrated the generation of polarization-entangled photon pairs using a 350-nm-thick 3R-WS_2_ crystal, achieving a CAR greater than 800 and a photon-pair generation rate of 31 Hz.

The enhanced performance of 3R-WS_2_ is further evidenced by the researchers’ ability to achieve robust quantum correlations at high pump powers, with the second-order correlation function g²(0) exceeding 800 at a pump power of 2.5 mW. In contrast, photon-pair sources based on 3R-MoS_2_ exhibit much lower g²(0) values at the same pump power level. At higher pump powers, the phenomenon is more noticeable, where photoluminescence noise becomes a significant issue.

These results are particularly impressive considering the spatial characteristic—compact size of the photon source. The 3R-WS_2_ crystal’s ability to support scalable optical nonlinearity while maintaining well-defined entanglement properties makes it an ideal candidate for integration with nanophotonic circuits. The high fidelity of the generated Bell states and fidelities exceeded 0.93, further underscoring the potential of this material for quantum applications.

The success of this experiment is attributed to the unique crystal symmetry of 3R-WS_2_, which enables a selection rule for SPDC that correlates the circular polarization states of the pump and emitted photons. This allows for the generation of maximally entangled Bell states in a controlled and efficient manner. Moreover, the high second-order optical susceptibility of 3R-WS_2_, enhanced by resonant coupling with electronic transitions, facilitates efficient photon-pair generation without introducing photoluminescence noise.

The successful demonstration of a high-performance polarization-entangled photon-pair source using 3R-WS_2_ represents a major step forward in the development of integrated quantum photonic systems. However, there are still challenges to overcome before these sources can be deployed in practical applications.

One of the key areas for future research is the integration of 3R-WS_2_ with resonant photonic structures, such as cavities and metasurfaces. These structures can enhance photon-pair generation rates by confining light and increasing the interaction length within the material. By combining 3R-WS_2_ with these structures, researchers could significantly boost photon-pair generation efficiency, closing the gap between vdW materials and traditional bulk nonlinear crystals. Another promising direction is the exploration of other vdW materials with similar or superior optical properties. By exploring different material systems and optimizing their integration with nanophotonic components, researchers can continue to push the boundaries of quantum photonic technologies.

In conclusion, the work of Feng et al. marks a significant advancement in the field of quantum photonics, demonstrating the potential of van der Waals materials like 3R-WS_2_ for generating high-quality, polarization-entangled photon pairs in a compact form factor. The ability to integrate these sources with nanophotonic structures opens new possibilities for quantum computing, communication, and sensing, bringing us closer to the realization of practical quantum technologies.

As the field continues to evolve, further research into vdW materials and their integration with photonic devices will be crucial for developing scalable quantum systems. The high performance of 3R-WS_2_ photon-pair sources, combined with the material’s compatibility with existing photonic architectures, positions it as a key player in the future of quantum photonics.
